# Spontaneous Resolution of Pharyngeal Myoclonus in a Child: An Uncommon Case With Objective Clicking Sounds

**DOI:** 10.7759/cureus.89518

**Published:** 2025-08-06

**Authors:** Koji Yokoyama, Mitsukazu Mamada

**Affiliations:** 1 Department of Pediatrics, Japanese Red Cross Wakayama Medical Center, Wakayama, JPN

**Keywords:** functional movement disorder, pediatric case, pharyngeal myoclonus, psychosocial stressors, spontaneous resolution

## Abstract

Pharyngeal myoclonus is a rare movement disorder characterized by rhythmic pharyngeal contractions, typically reported in adults with neurological lesions and rarely in children. We report a seven-year-old girl who experienced involuntary clicking sounds localized to the right ear. Nasopharyngoscopy revealed pharyngeal myoclonus without structural abnormalities, and brain and laryngeal MRI were unremarkable. No intervention was performed, and symptoms resolved spontaneously over 12 months, coinciding with increased familial support in response to suspected psychosocial stress. This case highlights the benign, self-limiting nature of pediatric pharyngeal myoclonus and suggests that conservative management may be appropriate, particularly when environmental or psychological factors are the suspected contributors.

## Introduction

Pharyngeal myoclonus is an uncommon neurological disorder characterized by involuntary rhythmic movements of the pharyngeal wall, often occurring in conjunction with palatal or laryngeal myoclonus. In adults, pharyngeal myoclonus is often linked to identifiable brainstem abnormalities, including ischemia and hypertrophic olivary degeneration, or metabolic conditions such as vitamin B12 deficiency. Brainstem lesions in the region of the Guillain-Mollaret triangle can cause disinhibition of the central tegmental tract and dentato-rubro-olivary pathways, resulting in hypertrophic olivary degeneration and rhythmic involuntary movements. Vitamin B12 deficiency, through its impact on myelin synthesis, may lead to demyelination of central nervous system structures, including the brainstem, and has been implicated in the pathophysiology of some myoclonic syndromes [1-4]. By contrast, pharyngeal myoclonus is extremely rare in children, with little known about its etiology or natural history in pediatric patients [5,6]. The present report describes a child with pharyngeal myoclonus who presented with externally audible “clicking” sounds. To our knowledge, this is one of the few documented pediatric patients to date presenting with endoscopically and audiologically confirmed pharyngeal myoclonus, which resolved spontaneously without intervention.

## Case presentation

A seven-year-old girl was referred for evaluation of intermittent, externally audible “clicking” sounds localized to her right ear. The phenomenon began during her final year of kindergarten, and psychosocial pressure associated with her senior-class role was suspected as a precipitating factor. By the time she entered the first grade of elementary school, the clicks persisted only on the right side, having resolved spontaneously on the left side. The sounds were involuntary, rhythmic, and disappeared during sleep or when she clenched her teeth. She denied otalgia, dysphagia, hoarseness, or any temporal relationship to swallowing or mastication. Her growth parameters were age-appropriate, and there were no signs of respiratory, nutritional, or neurological dysfunction. Family and peer relationships were stable, and her academic performance was satisfactory. Past medical and family histories were unremarkable. She had never been hospitalized, taken regular medications, or experienced allergic reactions. Physical examination showed intact bilateral tympanic membranes without effusion or retraction, with the clicks being clearly audible within 30 cm of her right ear (audio in Video 1).

**Video 1:** Clicking sounds synchronized with pharyngeal contractions Audio recording obtained during the initial consultation. Objective clicking sounds were perceived near the right ear and corresponded with pharyngeal contractions. The rhythmic and involuntary nature of the sound was evident, and the sounds could be heard clearly without amplification.

Flexible nasopharyngoscopy revealed irregular contractions at the naso-oropharyngeal junction synchronized with the audible clicks, with no mass lesions or structural abnormalities (Video 2).

**Video 2:** Endoscopic visualization of pharyngeal contractions synchronized with clicking sounds Flexible nasopharyngoscopy performed at the initial visit showed rhythmic, involuntary contractions at the junction of the nasopharynx and oropharynx. These movements corresponded precisely with the externally audible clicking sounds perceived near the right ear. No structural abnormalities or mass lesions are observed.

**Figure 1:**
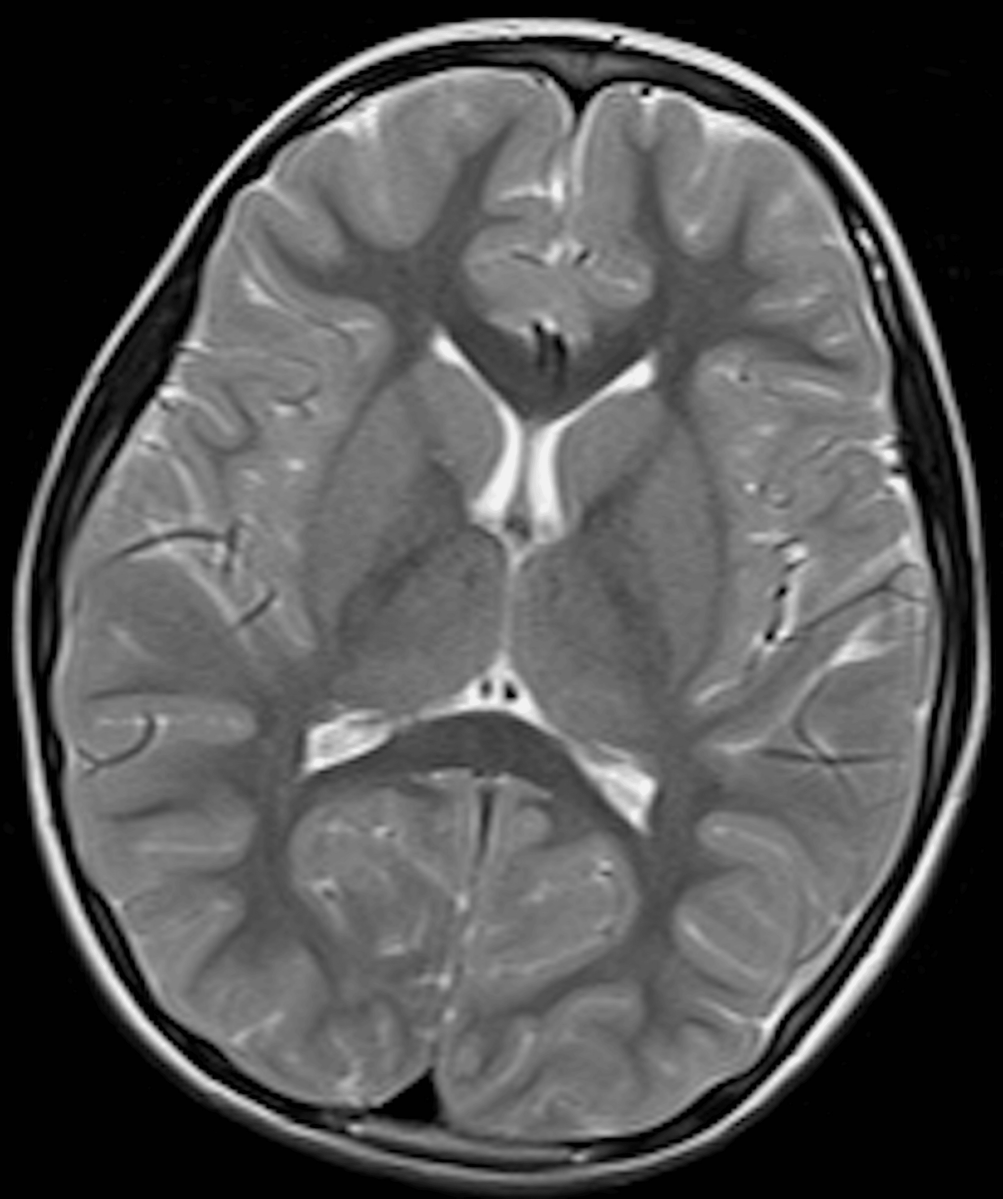
Normal axial T2-weighted brain MRI Axial T2-weighted MRI demonstrating normal cerebral anatomy at the level of the lateral ventricles. The image shows well-demarcated gray and white matter differentiation, normal ventricular size, and no evidence of mass effect, edema, or abnormal signal intensity. No structural abnormalities were observed.

**Figure 2:**
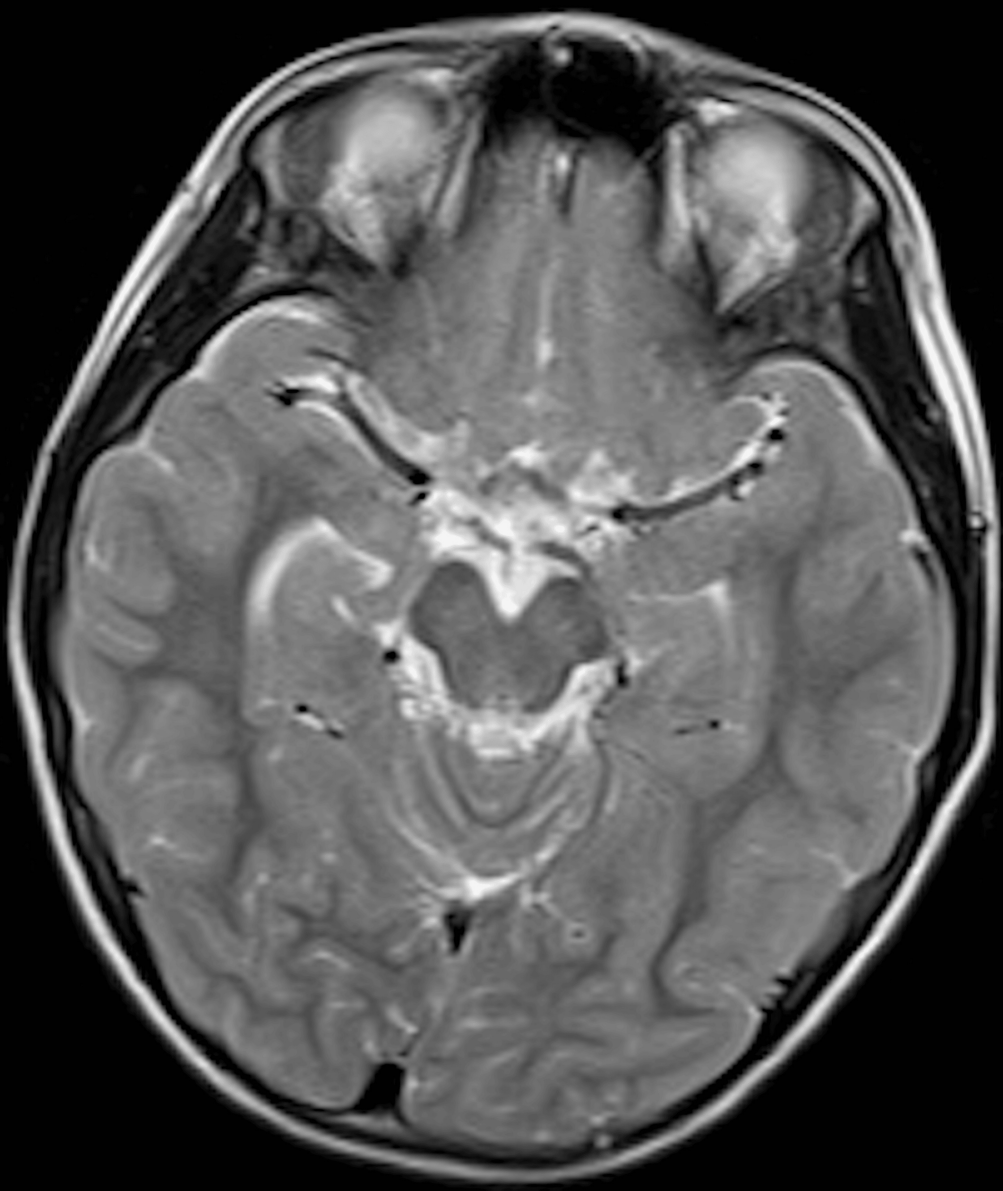
Normal brainstem on axial T2-weighted MRI Axial T2-weighted MRI at the level of the midbrain and pons. The brainstem structures, including the cerebral peduncles, midbrain tegmentum, and pontine base, appear symmetrical and without abnormal signal changes. No evidence of infarction, demyelination, or compression was observed  supporting the absence of brainstem pathology.

Neurological examination showed no evidence of cranial-nerve deficits or cerebellar signs. Ancillary investigations, including complete blood count, serum biochemistry with trace elements, throat culture, electrocardiography, electroencephalography, carotid ultrasonography, pure-tone audiometry, and brain and laryngeal magnetic resonance imaging (MRI), were all within normal limits; no brainstem or cerebellar lesions were identified (Figures 1, 2).

Following evaluation and interdisciplinary discussion involving pediatric, otolaryngology, and neurology teams, she was diagnosed with pharyngeal myoclonus. Owing to the benign, non-progressive course of this condition and the absence of underlying pathology, a watchful-waiting strategy was adopted after discussion with the patient and her family. During this period, the patient’s mother expressed concern that the symptoms might be stress-related, possibly triggered by emotional tension associated with school transition and family dynamics. In response, the family, including the patient’s parents and grandparents, engaged in open discussions about their interactions with the child and made efforts to provide a supportive environment. Although a direct causal relationship between these actions and the symptom resolution cannot be established, her myoclonus gradually subsided, and the patient adapted well to school life thereafter. During the 12-month follow-up period, the patient exhibited gradual resolution of pharyngeal myoclonus following the family’s efforts to provide emotional support. Although she occasionally developed upper respiratory symptoms, including sore throat associated with common viral infections, there was no worsening or recurrence of the clicking sounds. No pharmacological or surgical treatment was required throughout the follow-up period. This case report was reviewed and approved by the Ethics Committee of the Japanese Red Cross Wakayama Medical Center (approval number: 1502). Written informed consent was obtained from the patient’s parents for publication of this case report and accompanying audio and video materials.

## Discussion

Essential pharyngeal myoclonus is a rare neurological disorder characterized by involuntary movements of the soft pharyngeal musculature causing objecting-clicking tinning [1,4]. Pharyngeal myoclonus is most commonly encountered in adult patients, where it is often associated with lesions involving the Guillain-Mollaret triangle and hypertrophic olivary degeneration [3]. Pharyngeal myoclonus in adults generally requires pharmacological intervention, such as treatment with clonazepam, valproate, or gabapentin, and rarely resolves spontaneously [4,7]. 

Less is known, however, about pharyngeal myoclonus in pediatric patients. Palatal myoclonus has been reported in an eight‑year‑old, who showed spontaneous resolution of essential palatal myoclonus [5] and in a 12‑year‑old with clicking tinnitus attributed to palatal contractions [6]. These cases shared some clinical features with the present case, such as symptom pattern and a benign course, but myoclonus in these two patients involved the palatal rather than the pharyngeal musculature; moreover, objective endoscopic and audiologic documentation were lacking. 

To our knowledge, this is the first report of pharyngeal myoclonus in a child with both endoscopic and audio confirmation, no underlying structural or neurological abnormality, and complete spontaneous resolution. This pattern is similar to other pediatric neurological conditions, including benign childhood epilepsy, transient tics, and postural tremors, that frequently remit with neurodevelopmental maturation [8,9]. The absence of organic or structural findings, as shown by normal imaging, EEG, blood tests, and neuro-otologic evaluations, suggests a benign functional movement disorder. Several functional (psychogenic) movement disorders in childhood have been reported to present with stereotyped and involuntary motor symptoms that resolve as psychological stressors abate [10]. Although the present patient had no overt psychiatric history, subtle psychosocial stressors were present. For example, this child initially refused to attend school after entering the first grade, requiring parental accompaniment. Her interpersonal interactions within the family were also strained, including signs of emotional conflict with her sibling and difficulty forming bonds with grandparents. She was selective in whom she trusted among adults, suggesting underlying psychological sensitivity. While such stressors are not uncommon among school-aged children, the manifestation of these experiences as pharyngeal myoclonus is exceedingly rare. Thus, individual neurodevelopmental or psychological traits, such as heightened somatic reactivity or atypical stress processing, may have contributed to symptom expression in this patient.

Although increased familial support appeared to coincide with symptom improvement, the absence of standardized behavioral assessments or validated stress measures limits our ability to draw firm conclusions regarding causality. The relationship between psychosocial stressors and symptom expression thus remains speculative.

The findings in this patient indicate that not all movement disorders in children necessitate aggressive diagnostic or therapeutic intervention. If clinical and imaging findings are reassuring, a conservative and supportive approach may be sufficient. The absence of any anatomical lesions or metabolic abnormalities, together with normal MRI and EEG findings, suggests that this condition represented a benign functional disorder of the developing nervous system. The suppression of symptoms during sleep and the ability to transiently control the clicking also suggest a subcortical origin with modifiable motor output. Although the role of psychosocial stressors remains unclear, the resolution of symptoms following increased familial support suggests the potential influence of environmental and relational factors on symptom expression and course.

## Conclusions

This report describes a rare case of pharyngeal myoclonus in a child, presenting with audible clicking sounds and confirmed by endoscopic examination. The disorder resolved spontaneously over a 12-month period without pharmacological or surgical intervention. This case contributes to the limited literature on pediatric pharyngeal myoclonus and suggests that spontaneous remission may occur in the absence of identifiable structural or metabolic abnormalities. Conservative management may be a valid approach in similar patients, provided that serious pathology is excluded. While psychosocial factors may play a modulatory role in symptom expression, further structured assessment would be necessary to confirm their contribution to disease resolution.
